# Chronic pain in individuals who have visual impairments: a protocol for an international survey study

**DOI:** 10.3389/fpain.2025.1631770

**Published:** 2025-08-08

**Authors:** Jordi Miró, Ariadna Sampietro, Ester Solé, Pere Llorens-Vernet, Carlos Mora

**Affiliations:** 1 Universitat Rovira I Virgili, Unit for the Study and Treatment of Pain – ALGOS, Catalonia, Spain; 2 Department of Psychology, Research Center for Behaviour Assessment (CRAMC), Catalonia, Spain; 3 Institut d’Investigació Sanitària Pere Virgili, Universitat Rovira i Virgili, Catalonia, Spain

**Keywords:** chronic pain, blindness, visual impairment, online survey, pain management, health disparities

## Abstract

Individuals who have visual impairments (IVI) face unique challenges in coping with and adjusting to chronic pain. However, this population remains underrepresented in chronic pain research and often encounters barriers in accessing effective, tailored healthcare. The primary objective of this study is to generate baseline data on the prevalence, types, and impact of chronic pain in this population. The secondary objectives are to identify specific challenges in pain management and provide evidence to identify specific challenges in pain management and to provide evidence that can inform the development of targeted interventions aimed at promoting equitable healthcare and improving quality of life. An international cross-sectional observational study will be conducted using an online survey administered via LimeSurvey. The survey, developed specifically for this project, will be accessible in multiple languages to enhance participation and representativeness. It is designed to capture the unique aspects of chronic pain, ocular and non-ocular forms, in IVI, including barriers to care and management challenges. This protocol aims to establish a comprehensive understanding of chronic pain in IVI. The findings will help bridge current research gaps, guide tailored interventions, and inform policy initiatives, ultimately reducing healthcare disparities and enhancing quality of life for this underserved group.

## 1 Introduction

Chronic pain has been defined as a problem that last 3 months or longer (1). It is a highly prevalent condition (2–4) that significantly impacts the individual, family and society (5–7). For individuals who are blind or who have low vision (henceforth referred to as IVI), chronic pain presents unique challenges, yet this population remains underrepresented in pain research. However, the scant data available show that chronic pain in this population is prevalent (8). Moreover, it has some specific characteristics that may result in a higher risk of developing chronic pain and related disability. This includes physical, psychological and social factors. For example, blindness can lead to changes in brain plasticity (9). In some cases, the reorganization of sensory processing areas in the brain may influence the perception and intensity of pain (10), thus increasing the risk of developing chronic pain problems. For example, Touj and colleagues (11) demonstrated that anophthalmic mice exhibit heightened pain sensitivity during the formalin test, along with increased c-Fos expression and larger amygdala volumes. Although most neuroplasticity findings and altered pain thresholds have been explicitly reported in people with congenital blindness, these findings suggest that the absence of visual input may enhance pain sensitivity through central mechanisms, warranting further exploration in human populations who have visual impairments. In parallel, other studies show that chronic pain itself can alter sensory processing. Bultitude and colleagues (12), for example, found that patients with complex regional pain syndrome exhibited spatial bias in visual attention, suggesting a bidirectional association between pain and sensory systems. These findings support the hypothesis that visual impairment and chronic pain may interact through shared neuroplastic mechanisms.

Beyond neuroplasticity, this population often adopt altered movements and postures to navigate their environment (e.g., leaning forward to use tactile cues which can strain the neck and back) or reduce their physical activity, leading to muscle weakness and reduced flexibility, or even overuse certain muscle groups (e.g., when using a cane or guide dog). These changes and compensations can contribute to chronic musculoskeletal strain. In addition, this group has been shown to experience elevated levels of anxiety and depression (13) and social isolation (14), which have been associated to the development of chronic pain (15, 16) and worse quality of life (17, 18).

Individuals who have visual impairments often face challenges accessing appropriate healthcare, including pain management resources and preventive care. Many healthcare systems often rely heavily on visual forms of communication, such as printed forms, digital interfaces, or signage in clinics and hospitals, that may not be accessible to IVI. Additionally, most health professionals lack proper training in how to interact with and treat this population, which contribute to disparities in diagnosis, treatment and outcomes (19, 20). In fact, research has shown that individuals with disabilities, including visual impairments, report worse health outcomes and greater difficulty accessing care compared to the general population (21).

Despite these intersecting challenges, limited data exists to inform tailored interventions for chronic pain management in this population (8). Existing surveys on chronic pain typically focus on general populations or specific clinical conditions, overlooking the lived experiences and contextual factors of IVI. For example, factors such as access to accessible pain management resources, barriers to physical therapy, and the role of social support systems for this population are rarely included in standard survey designs. The lack of inclusive research perpetuates disparities in healthcare access and outcomes for this population.

## 2 Objectives

The long-term goal of this project is to better understand chronic pain in IVI, with the aim of reducing its impact on their lives. To this end, we propose the development and implementation of a comprehensive cross-sectional survey for several key purposes. The primary objective of this study is to generate baseline data on the prevalence, types, and impact of chronic pain in this population. The secondary objectives are to identify specific challenges in pain management and to generate evidence that can inform the development of targeted interventions aimed at promoting equitable healthcare and enhancing quality of life.

## 3 Materials and methods

### 3.1 Study design

This is an international observational cross-sectional study.

### 3.2 Survey procedure/administration

A survey is conducted online using LimeSurvey, a web-based survey platform (https://www.limesurvey.org/). LimeSurvey was selected because it complies with WCAG 2.1 accessibility standards, ensuring compatibility with screen readers and keyboard navigation. Participants can also choose to respond using structured phone interviews or voice-assisted digital tools, depending on their preferences and access needs. The survey is available in multiple languages to increase participation and representativeness of the findings. The survey items have been specifically developed for this project.

The survey takes an average of less than 20 min to complete (no responses will be excluded based on the time taken to complete the survey). Once participants consent to participate in the study (after reading the participant's information sheet, that will be available through an external link), they are sent directly to the survey which has 17 questions included in 3 sections: Demographics (i.e., gender, age, country of residence, education, and employment status), Vision-related issues (e.g., “please, identify the cause of your blindness or low vision”), and Pain-related issues (e.g., “during the past 3 months have you had pain?”). There is a final, fourth section, that invites participants to indicate their potential interest in collaborating on future studies. Participants are given the option to provide their email address if they wished to be contacted for future research opportunities. Participants who do not provide an email address will remain completely anonymous. In addition, we offer the possibility to respond via structured phone interviews or accessible voice-assisted digital tools.

#### 3.2.1 Survey development and validation

The survey instrument was developed following an extensive literature review and through consultations with experts in pain research, pain management, disability studies, and survey methodology. Initial survey items were constructed based on the findings from previous research and recommendations from domain experts. As the original version of the survey was developed in Catalan, its preliminary version was pilot tested with three Catalan speaking IVI to assess clarity, accessibility, and cultural relevance. Feedback from these pilot sessions was used to refine the wording, format, and content of the questions to ensure that the survey is both reproducible and replicable. While this protocol paper does not include psychometric validation of the survey instrument -given its descriptive purpose and the nature of the data collected- future publications based on the study dataset will report psychometric properties where applicable. This will help ensure transparency and support the appropriate interpretation of the results in subsequent analyses.

#### 3.2.2 Survey translation and linguistic validation

To ensure the survey's accessibility on an international scale, the instrument has been translated into several languages, including English, French, Portuguese (Portugal) and Spanish. For each language version, the survey was first translated by an independent bilingual expert and then back-translated into the original language by a separate translator. Discrepancies between the original and back-translated versions were resolved through discussion among the research team and external linguistic consultants. Pilot testing was conducted in each language version with 3–4 members of the target population to verify that the items were culturally appropriate and clearly understood. However, the objective is to expand the number of available language versions to further increase accessibility and enhance international reach. Additional translations are currently in development, and all new versions will follow the same rigorous process of translation, back-translation, and pilot testing to ensure consistency and cultural validity. Thus, the final list of participating countries will depend on the successful completion and validation of these translations, as well as the availability of local dissemination channels. To support broad international representation, we are collaborating with organizations and professionals in multiple regions. Survey administration will be conducted remotely via accessible online platforms or, when needed, through structured interviews conducted by trained personnel.

### 3.3 Participant inclusion criteria

People that are 18 years of age or older and who have visual impairments are invited to participate. In order to participate, participants are requested to provide their informed consent. In line with widely accepted definitions (1) and prior epidemiological research (4, 22, 23), chronic pain in this study is defined as pain that has persisted for a minimum of three months and occurred at least once per week during that timeframe. Adopting this operational definition allows for comparability with findings from other well-established studies in the field.

### 3.4 Participants' recruitment

We have approached the existing blind associations that are part of the World Blind Union (https://worldblindunion.org/) and requested their help. They all have received an email describing the study, the procedure and objectives. In that message, we asked them to help us reach their associates and share the link to the survey study. The survey is disseminated globally with the help of the interested organizations. Moreover, we are also utilizing snowball sampling, peer-to-peer referrals, and social media dissemination to reach individuals who may not be affiliated with formal disability organizations. No incentive is provided for their participation.

To ensure reliable survey data, we block duplicate entries by restricting multiple accesses from the same IP address. In addition, we will manually review the dataset to remove any duplicates by comparing the demographic information provided by participants. Two researchers will independently check all entries for duplicates, and a third reviewer will resolve any discrepancies if needed.

The required sample size was estimated based on an expected prevalence of chronic pain of 20%, as shown by international epidemiological studies with adults (24). Assuming a 95% confidence level (*Z* = 1.96) and a precision of ±5% (*d* = 0.05), the sample size was calculated using the standard formula: *n* = [*Z*^2^ × *P* × (1–*P*)]/*d*^2^, which means a total of 245.86 participants [*n* = (1.96)^2^ × 0.20 × (1–0.20)/(0.05)^2^ = 3.8416 × 0.16/0.0025 = 245.86]. To account for an anticipated attrition rate of 15%, the adjusted sample size is: *n*-adjusted = 246/(1–0.15) = 289.4. Therefore, the target recruitment goal is set at 290 participants, ensuring that the study is sufficiently powered to estimate the prevalence of chronic pain with a 95% confidence level, 5% margin of error, and adequate compensation for potential data loss. We aim to recruit up to 290 participants in each participating country. However, we are aware that reaching this target may be challenging in some contexts. This limitation is anticipated and will be acknowledged as one of the practical constraints of the project. Data collection is expected to be completed by August 2025, with the results reported before the end of 2025. However, data collection will remain open until the target number of participants is reached or until it becomes evident that further extension of the recruitment period would not yield a substantial increase in responses. This flexible approach is intended to accommodate variations in recruitment pace across countries and ensure data quality without unnecessarily prolonging the study timeline.

### 3.5 Data analysis

We will use descriptive statistics to describe the sample and the study variables (i.e., means, standard deviations, and percentages). In addition, we will conduct stratified and sensitivity analyses to explore variability within the sample. These analyses will examine differences in chronic pain prevalence and characteristics according to the following variables: type of visual impairment (congenital vs. acquired), severity of vision loss, underlying cause of the impairment, and pain severity. Given the international nature of the study, we will also explore associations between country-level characteristics (e.g., healthcare accessibility) and participants' reported pain and barriers to care. While these subgroup analyses will be exploratory, they may help identify disparities that warrant further investigation.

### 3.6 Ethics and consent

The Ethical Committee concerning Research into People, Society and the Environment (CEIPSA) of the *University Rovira i Virgili* approved the study procedures (ref.: CEIPSA-2021-PR-0043). Potential participants will be required to provide informed consent either in written form (by ticking a checkbox in the same online form) r verbally, before completing the survey. If they participate in a telephone survey form, their verbal consent will be recorded. Once they consent to participate, they will be allowed to enter the survey website, read the questions, and respond. The protocol has been registered in OSF (25) to ensure transparency and reproducibility.

### 3.7 Follow-up interviews

To complement the survey data and gain deeper insight into participants' experiences with chronic pain, a subset of 10 participants will be invited to participate in in-depth, semi-structured interviews. The sample size of 10 participants was selected based on thematic saturation guidelines commonly applied in qualitative pain research (26, 27).These interviews aim to further explore pain characteristics, challenges in accessing treatment, and coping strategies, and will also help identify potential reporting biases in the survey responses. Participants who indicate their willingness to be contacted for future studies (via the final section of the survey) will be considered for this phase. Interviews will be conducted remotely via telephone or secure online platforms, depending on participants’ preferences and accessibility needs. All interviews will be audio-recorded with informed consent and transcribed verbatim. A thematic analysis approach will be used to analyze the data, allowing for the identification of common patterns and unique perspectives that may not be fully captured through quantitative survey responses.

## 4 Discussion

Chronic pain is a pervasive issue affecting a significant portion of the global population, leading to substantial personal and societal burdens. While extensive research has been conducted on chronic pain in the general population, there remains a notable gap in understanding its prevalence, characteristics, and management among IVI. This study protocol aims to address this gap by outlining a comprehensive survey designed to investigate chronic pain experiences in this underserved population.

### 4.1 Importance of the study

While neuroplastic changes and altered pain perception have predominantly been documented in individuals with congenital or early-onset blindness, emerging evidence indicates that altered sensory integration and compensatory mechanisms in the broader population of individuals with visual impairments may modulate pain processing in distinct ways. The absence or reduction of visual input can lead to increased reliance on other senses, potentially resulting in heightened sensitivity or altered pain thresholds (28). Studying this population will provide valuable insights into the neural mechanisms underlying pain perception and its modulation. While the survey does not directly assess physiological mechanisms of pain modulation, it will provide self-reported data that may suggest perceptual differences requiring further investigation. Research suggests that attentional biases to painful stimuli are evident in individuals with chronic pain (29), indicating that sensory processing plays a crucial role in pain perception. However, the directional tendency of these biases (i.e., toward or away from threat-related stimuli) remains unclear, necessitating further research in populations who have visual impairments. Moreover, these individuals often adopt postures or engage in repetitive movements that can lead to musculoskeletal strain, potentially increasing the risk of developing chronic pain conditions.

### 4.2 Necessity of the study

The necessity of this study is underscored by the limited availability of pain assessment tools validated for use in IVI. Traditional pain assessment scales often rely on visual cues, making them less accessible to those with visual disabilities. Research has shown that healthcare providers may not be adequately trained in assessing and managing pain in IVI (20), leading to missed opportunities for early identification and treatment. By providing data to help in developing and validating accessible and reliable pain assessment tools specifically designed for IVI, this study addresses a critical gap in current healthcare practices. Furthermore, a better understanding of the specific challenges faced by IVI and chronic pain could lead to the development of more targeted and effective treatment strategies. This study will also identify specific barriers to pain management, allowing for the development of strategies to overcome them and improve overall healthcare accessibility for this population. Another key necessity of this research is its potential to inform public health policies. By generating evidence-based insights, policymakers can develop regulations and guidelines to ensure that healthcare services for these individuals incorporate specialized pain assessment and treatment protocols. This is especially important given the growing recognition of chronic pain as a public health concern that requires systematic approaches for prevention and management (30). In addition, the global scale of visual impairment highlights the urgency of addressing this issue. According to the World Health Organization (https://www.who.int/news-room/fact-sheets/detail/blindness-and-visual-impairment), more than 2.2 billion people worldwide have some form of vision impairment, and the number of individuals living with blindness is projected to reach 115 million by 2050. These figures emphasize the importance of ensuring equitable access to pain assessment and management resources for this large and growing population.

### 4.3 Impact for the future

Findings from this study have the potential to significantly impact future research, clinical practice, and policy development. By elucidating the specific chronic pain experiences of IVI, this research can inform the development of targeted pain management interventions that are accessible and effective. This study will provide valuable insights into the interplay between sensory, cognitive, and emotional factors that contribute to the experience of chronic pain in IVI, enhancing our understanding of the mechanisms underlying chronic pain. In addition, the study's results could guide the adaptation of existing pain assessment tools or the creation of new ones that accommodate the needs of IVI. Moreover, by raising awareness among healthcare providers about the unique pain experiences in this population, this research advocates for improved healthcare practices and policies. Ensuring that healthcare professionals receive adequate training in assessing and managing pain in IVI is essential for equitable and effective treatment. Furthermore, this project has implications beyond the group of IVI and chronic pain. That is, findings may contribute to general pain research by identifying potential therapeutic targets applicable to broader populations. Understanding pain perception in individuals with sensory impairments could lead to innovations in pain management strategies that benefit a wide range of patients. From a technological perspective, this research could drive innovation in assistive technologies aimed at pain assessment and management for individuals with visual impairments who have chronic pain. For example, voice-activated, tactile-based or artificial-intelligence-assisted pain assessment tools could be developed to enhance self-reporting accuracy.

### 4.4 Expanding the scope of pain management for individuals who have visual impairments

A major challenge for IVI and chronic pain is the lack of accessible self-management strategies. Pain management programs commonly emphasize self-monitoring, guided exercises, and relaxation techniques that often require visual instruction. Developing adaptations of these programs tailored to individuals with visual impairments could significantly improve their effectiveness. For instance, audio-based pain management programs and tactile instructional tools could empower IVI to engage more actively in their pain management.

Moreover, psychological interventions play a crucial role in chronic pain management. Cognitive-behavioral therapy (CBT) has been widely used to help individuals with chronic pain (31), but existing models may need adaptation for this population. Creating specialized CBT programs that account for sensory differences and unique coping strategies among IVI would enhance their accessibility and effectiveness.

In addition, interdisciplinary care models that integrate pain specialists, occupational therapists, and rehabilitation professionals trained in working with IVI could improve treatment outcomes. Given the complex interplay between sensory processing, emotional regulation, and chronic pain, a multidisciplinary approach is essential for providing comprehensive care to IVI and chronic pain.

### 4.5 Advocacy and policy development

The findings from this project can be used to advocate for policies that mandate training programs for healthcare providers, ensuring that pain assessment and management strategies are inclusive of IVI (32). Another critical aspect of advocacy is increasing public awareness about the impact of chronic pain on IVI. Educational campaigns can help reduce stigma and ensure that IVI receive the support and accommodations necessary for effective pain management. Nonprofit organizations and advocacy groups can use the study's findings to push for systemic changes that promote equity in pain treatment and rehabilitation services.

### 4.6 Limitations

Despite its strengths, this study on chronic pain in IVI may encounter several challenges and limitations. First, the recruitment strategy relies mainly on organizations for IVI, which may result in a sample that is not fully representative of the population who have visual impairments and chronic pain. Moreover, those who are actively engaged in such organizations might have better access to healthcare, resources, or support systems, which could influence their pain experiences compared to those who are not affiliated with these networks. Additionally, individuals with severe disabilities or comorbid conditions may be underrepresented due to difficulties in participation. However, this survey will be conducted across different countries, therefore ensuring representation of diverse demographics (e.g., age, gender, socioeconomic status, level of visual impairment) by actively recruiting from different regions and community settings. In addition, we will encourage enrolled participants to refer peers with similar conditions could help include individuals who may not be affiliated with formal organizations. In relation to this, another anticipated limitation of this study is the potential variability in recruitment success across participating countries. While we aim to recruit approximately 290 participants per country to allow for meaningful country-level analyses, we recognize that this target may not be feasible in all settings due to differences in population size, organizational reach, and resource availability. To address this, we will adopt a flexible recruitment strategy, leveraging both formal networks (e.g., national associations of persons with visual impairments) and informal channels (e.g., social media, peer referrals) to maximize participation. In cases where country-specific samples are smaller than expected, data will still be included in the overall analysis, and sample size limitations will be transparently reported and considered in the interpretation of results. Moreover, although the study aims to explore variability within the population of IVI, including subgroup analyses based on type of visual impairment, and pain severity, these analyses may be limited by sample size constraints. As the survey is conducted across multiple countries and relies on volunteer participation, certain subgroups (e.g., individuals with congenital blindness or those from lower-income regions) may be underrepresented. Consequently, some stratified or sensitivity analyses may lack sufficient statistical power, and their findings should be interpreted with caution. Nonetheless, these exploratory comparisons are expected to provide valuable preliminary insights and help inform future, more targeted studies.

Second, to maximize completion rates, we intentionally kept the survey brief, which limited the amount of information being collected. Consequently, our ability to fully interpret and contextualize respondents' answers is constrained. Third, the study relies exclusively on self-reports to assess pain and its impact, which introduces potential biases, such as recall bias. While the assessment tools will be adapted for individuals with visual impairments, there may still be challenges in ensuring that participants interpret and respond to pain measures consistently over time. However, we will conduct pilot testing with individuals with visual impairments to ensure that survey adaptations are clear, intuitive, and effectively capture pain experiences. In addition, we will provide participants with the option to use structured phone interviews or accessible voice-assisted digital tools. This approach ensures that all participants interpret the questions similarly and report their pain symptoms consistently. Moreover, we will conduct in-depth interviews with a subset of participants to complement survey data with a deeper understanding of their pain experiences and potential reporting biases that will also be of help to investigate the reliability of the data.

Fourth, although the study will implement accessible survey formats and offer telephone interviews when needed, differences in participants' technological literacy or communication preferences may still pose challenges. Some individuals may face difficulties using online surveys, while others may not feel comfortable sharing detailed pain experiences in a phone interview. Ensuring that all participants have equal opportunities to provide accurate responses remains a key consideration. In order to address this issue, we will involve IVI in the development and testing of survey formats to ensure usability and clarity, provide the survey in multiple accessible formats, including screen-reader-compatible web forms, braille materials upon request, and audio-recorded versions, offering a helpline or email support system for participants facing difficulties with the online survey platform and ensuring that researchers conducting phone interviews are trained in best practices for communicating with individuals with visual impairments, including patience, clarity, and structured questioning.

Fifth, obtaining informed consent from participants who have visual impairments requires careful attention to accessibility. Although accessible formats will be provided, variations in participants' literacy levels (both general and digital) could impact their ability to fully understand study procedures. Ensuring that consent materials are clear, comprehensible, and adapted to different needs is crucial for ethical study conduct. Allowing participants to provide verbal consent during recorded phone calls, ensuring they fully understand the study's purpose and procedures will help ensuring that participants fully grasp the study's requirements and their rights.

Sixth, another limitation of this study is the potential presence of duplicate responses. Although we implement technical measures to block multiple submissions from the same IP address and conduct manual reviews to identify duplicates based on demographic and response patterns, it is not possible to guarantee complete elimination of duplicate entries. The manual review process involves two independent researchers and a third reviewer to resolve discrepancies; however, the lack of uniquely identifying information limits the certainty of this approach. As such, some duplicate responses may remain undetected, or conversely, valid responses may be inadvertently excluded.

### 4.7 Conclusion

Despite potential limitations, this study protocol represents an important step toward understanding and managing chronic pain in individuals with visual impairments. By exploring the prevalence, characteristics, and management of chronic pain in this population, the findings will fill a significant gap in the current literature. The anticipated findings hold promise for informing future interventions, assessments, and policies, ultimately contributing to improved health outcomes and quality of life for IVI and chronic pain. Furthermore, this research has the potential to drive innovations in assistive technology, expand the scope of tailored pain management strategies, and advocate for systemic changes in healthcare and policy. By addressing these gaps, this study will help pave the way for a more inclusive and effective approach to chronic pain management.

## Data Availability

The original contributions presented in the study are included in the article/Supplementary Material, further inquiries can be directed to the corresponding author.
