# Correction: Chronic pain in individuals who have visual impairments: a protocol for an international survey study

**DOI:** 10.3389/fpain.2026.1868545

**Published:** 2026-05-14

**Authors:** Jordi Miró, Ariadna Sampietro, Ester Solé, Pere Llorens-Vernet, Carlos Mora

**Affiliations:** 1 Universitat Rovira I Virgili, Unit for the Study and Treatment of Pain – ALGOS, Catalonia, Spain; 2 Department of Psychology, Research Center for Behaviour Assessment (CRAMC), Catalonia, Spain; 3 Institut d’Investigació Sanitària Pere Virgili, Universitat Rovira i Virgili, Catalonia, Spain

**Keywords:** chronic pain, blindness, visual impairment, online survey, pain management, health disparities

The funder Spanish Ministry of Science, Innovation and Universities, FPU predoctoral fellowship FPU23/03355 to Ariadna Sampietro was erroneously omitted.

The corrected Funding statement appears below:

“The author(s) declare that financial support was received for the research and/or publication of this article. This study is partially supported by a grant from the Spanish Ministry of Science and Innovation (ref. RED2022-134869-T); the European Regional Development Fund (ERDF); and the Government of Catalonia (AGAUR; 2021SGR-730). JM's work is supported by ICREA-Acadèmia. AS is supported by a FPU predoctoral fellowship from the Spanish Ministry of Science, Innovation and Universities (FPU23/03355). The Chair in Pediatric Pain is supported by Fundación Grünenthal and ANUBIS.”

The original version of this article has been updated.

